# Neural network-driven analysis of magnetized dissipative Ree-Eyring fluid flow with Cattaneo-Christov heat flux on a permeable surface

**DOI:** 10.1186/s11671-026-04815-z

**Published:** 2026-08-03

**Authors:** Ebrahem A. Algehyne, Safa Elshaikh Saad Ahmed, Muntasir Suhail, Fahad Maqbul Alamrani, Anwar Saeed, Gabriella Bognár

**Affiliations:** 1 Department of Mathematics, Faculty of Science, University of Tabuk, P.O. Box 741, 71491 Tabuk, Saudi Arabia; 2 Department of Mathematics, Al Khurmah University College, Taif University, P.O. Box 11099, 21944 Taif, Saudi Arabia; 3 Department of Mathematics, College of Science, Qassim University, 51452 Buraydah, Saudi Arabia; 4 Department of Computer Engineering, Biruni University, 34010 Istanbul, Turkey; 5 Institute of Machine and Product Design, University of Miskolc, Miskolc- Egyetemváros, 3515 Hungary

**Keywords:** Magnetohydrodynamic (MHD), Ree-Eyring fluid, Cattaneo-Christov heat flux model, Stretching surface, Darcy-Forchheimer porous medium, Thermal radiation

## Abstract

This work investigates the magnetohydrodynamic flow of a dissipative Ree-Eyring fluid over a permeable stretching surface, subjected to an inclined magnetic field relative to the direction of fluid motion. Thermal transport is analyzed using the Cattaneo-Christov heat flux model, which accounts for thermal relaxation effects absent in the classical Fourier formulation. The flow regime further incorporates the influence of a Darcy-Forchheimer porous medium, introducing both linear and quadratic drag contributions to the momentum equation. The main equations have initially evaluated numerically through bvp4c approach in dimensionless form. The dataset generated through the bvp4c numerical scheme is subsequently employed to implement the artificial neural network (ANN) methodology. It has revealed as outcomes of this work that optimal convergence achieved through ANN approach at epochs 134, 205, and 275 across the three scenarios. Error histograms and fitness evaluation confirm solution stability, progressive improvement, and close alignment between predicted and expected values. With growth in Weissenberg number $\left( {We} \right)$, magnetic parameter $\left( M \right)$, Darcy Forchheimer factor $\left( {Fr} \right)$ and porosity parameter $\left( K \right)$ there is decline in velocity $\left\{ {f^{\prime}\left( \eta \right)} \right\}$. Thermal distribution $\left\{ {\theta \left( \eta \right)} \right\}$ augmented with growth in radiation parameter $\left( {Rd} \right)$, Brownian motion parameter $\left( {Nb} \right)$, thermo-phoresis parameter $\left( {Nt} \right)$, heat source/sink factor $\left( Q \right)$ while declined with higher Prandtl number $\left( {\Pr } \right)$ and thermal relaxation time parameter $\left( \delta \right)$.

## Introduction

 Magnetohydrodynamics (MHD) is a fascinating interdisciplinary study of the motion of electrically conductive fluids usinf magnetic fields, encompassing phenomena that span from astrophysical scales to microfluidic devices. The fundamental principle underlying MHD lies in the reciprocal interaction between the velocity field and the electromagnetic field: the motion of conducting fluid across magnetic field lines induces electric currents, which in turn generate Lorentz forces that modify the original flow pattern. Abdal et al. [1] discussed magnetohydrodynamics fluid flow with Cattaneo-Christov heat flux using machine learning approach. Farooq et al. [2] studied magnetohydrodynamics stagnant point flow of nanofluid on surface with nonlinear radiative effects. Algehyne et al. [3] examined magnetized nanofluid flow with thermal and flow slip effects on a varying porous surface. The relative importance of magnetic effects to inertial effects is quantified by the magnetic Reynolds number, while the Alfvén velocity represents the characteristic speed at which magnetic disturbances propagate through the conducting medium. Reddy et al. [4] examined numerically the production of entropy for magnetized stagnant point flow of nanofluid with activation energy effects. In industrial applications, MHD principles enable contactless flow control in metallurgical processes, where electromagnetic stirring enhances alloy homogeneity and electromagnetic braking regulates molten metal flow in continuous casting operations. The aerospace sector leverages MHD for hypersonic vehicle thermal protection, where magnetic fields manipulate ionized shock layers to reduce heat transfer rates. Kalita and Dey [5] studied the substantial effects of slip constraints with dissipative impacts on a time-dependent magnetohydrodynamics fluid flow on a medium. Magnetohydrodynamic generators in energy systems offer direct conversion of thermal energy to electricity without moving mechanical components, while fusion reactor designs rely on magnetic confinement to sustain plasma stability. Waqas et al. [6] discussed mixed convective micro-polar magnetohydrodynamic fluid flow on a nonlinear stretchable surface with convective constraints. The presence of magnetic fields fundamentally alters boundary layer characteristics, suppressing velocity profiles through Lorentz retardation while simultaneously generating Joule heating that modifies thermal transport. Hayat et al. [7] studied magnetized thermally radiative fluid flow on a stretchable surface with heat source and chemically reactive effects by implementing the neural network approach. Mandal and Pal [8] examined radiative magnetized nanofluid flow on Darcy-Forchheimer permeable surface. The continued advancement of MHD research promises innovations in energy conversion, materials processing, and biomedical therapeutics, driven by deeper understanding of magneto-fluid interactions across diverse length and time scales.

The flow of Ree-Eyring fluid describes a fascinating departure from the Newtonian paradigm, offering a mathematical model that captures the complex, non-linear relationship between stress and deformation rate exhibited by many real-world materials. Unlike simple fluids where viscosity remains constant, Ree-Eyring fluids belong to the class of generalized Newtonian fluids, characterized by a viscosity that varies with the shear rate. This specific model [9], an extension of the simpler Eyring model, posits that the fluid’s viscosity decreases as the shear rate increases, a phenomenon known as shear-thinning. Naeem et al. [10] analyzed Ree-Eyring fluid flow between two narrow disks, incorporating temperature-dependent viscosity. Their study explores how thermal variations influence the fluid’s shear-thinning behavior, providing crucial insights into heat transfer and flow dynamics in confined geometries relevant to industrial lubrication and cooling systems. The elegance of the Ree-Eyring model lies in its incorporation of multiple relaxation times or activation energies, allowing it to more accurately describe the flow over a broader range of shear rates than its predecessor. This behavior arises from the molecular or microstructural nature of the fluid; as stress is applied, the constituent molecules or particles align with the flow, reducing internal resistance. This makes it particularly adept at modeling the behavior of complex fluids like polymer melts, lubricants under extreme pressure, and even biological fluids [11–13]. In a lubrication context, for instance, as surfaces move at high speeds, the lubricant experiences immense shear rates. Abbas et al. [14] discussed the production of entropy for Ree-Eyring fluid flow through a permeable channel using several constraints. A Ree-Eyring model predicts that the lubricant’s viscosity will drop in this high-shear region, a critical factor in calculating film thickness, friction, and ultimately, the efficiency and durability of mechanical components like gears and bearings. Mathematical formulation introduces non-linear terms into the governing equations of fluid dynamics, making analytical solutions a rarity and often necessitating sophisticated numerical methods for analysis. By accounting for the yielding and subsequent flow of microstructural elements under stress, the Ree-Eyring constitutive equation provides engineers and scientists with a powerful tool to design better systems, predict material behavior under extreme conditions. Abdullah et al. [15] studied the thermal transference for Ree-Eyring fluid flow with effects of magnetic field. Reddy et al. [16] studied the simulation of Darcy-Forchheimer magnetized Ree-Eyring fluid flow on a surface with microorganisms and injection/ suction effects.

The incorporation of the Cattaneo-Christov heat flux model into the analysis of fluid flow represents a substantial theoretical advancement over the classical Fourier’s law, addressing the paradox of infinite thermal propagation speed that has long challenged conventional heat transfer analysis. While Fourier’s law [17] assumes that any thermal disturbance propagates instantaneously through the medium, the Cattaneo-Christov model [18, 19] introduces a crucial relaxation time parameter that accounts for the finite speed of thermal waves, thereby capturing the subtle reality of heat propagation in materials with non-homogeneous inner structures. This refined constitutive relationship modifies the classical heat equation by incorporating a thermal inertia term, transforming the parabolic energy equation into a hyperbolic one that admits wave-like heat propagation characteristics. Hayat et al. [20] studied Jeffrey fluid flow on a stretchable surface with Cattaneo-Christov thermal flux effects subject to chemical reactivity. When applied to flowing fluids, particularly those exhibiting complex rheological behavior, the Cattaneo-Christov framework reveals fascinating interactions between momentum transport and thermal energy distribution. The model introduces a material derivative of the heat flux, effectively building in a memory effect whereby the fluid’s thermal state depends not merely on instantaneous temperature gradients but also on its thermal history and the convective motion of the medium. This becomes especially significant in boundary layer flows, where the scenario between fluid motion and thermal relaxation can lead to delayed thermal response, altered temperature profiles, and modified heat transfer rates at solid boundaries. Malleswari et al. [21] studied thermally the radiative magnetized Darcy-Forchheimer fluid flow with Cattaneo-Christov flux theory effects on a surface with activation energy. The mathematical implementation introduces additional non-linear terms into the energy equation, coupling the temperature field with velocity components in ways that classical Fourier analysis cannot capture. For engineering applications, these effects prove particularly relevant in processes involving extremely high temperature gradients, rapid heating or cooling, microscale heat transfer, and flows of polymeric or nano-structured fluids where the internal relaxation times become comparable to flow time scales. Khan et al. [22] studied Jeffrey nanfluid flow and optimization of entropy with Cattaneo-Christov thermal flux effects by implementing the neural network approach. Ravi Kumar et al. [23] examined magnetized nanofluid flow with Cattaneo-Christov thermal flux effects on a permeable elongating surface with microorganisms’ effects. Razzaq et al. [24] investigated dual diffusive nanofluid flow and optimization of entropy on a surface with Cattaneo-Christov fluxes. Researchers have increasingly applied this sophisticated model to study stagnation point flows, stretching surfaces, rotating disks, and peristaltic transport, revealing that the thermal relaxation parameter can significantly delay thermal boundary layer development and reduce surface heat flux compared to Fourier predictions. The Cattaneo-Christov framework thus bridges the gap between classical continuum heat transfer and the growing recognition that thermal energy transport in complex materials requires consideration of both spatial and temporal non-localities.

The study of fluid flow on a stretching surface constitutes one of the most industrially relevant and mathematically rich areas of fluid mechanics. When a sheet emerges from a slit and is stretched through a quiescent or moving fluid, the boundary layer that develops along its surface exhibits fascinating characteristics that differ fundamentally from flow over rigid bodies. The stretching motion itself induces fluid motion through viscous entrainment, creating a self-similar velocity field that has captivated researchers since the pioneering work of Crane in the early 1970s [25]. Sohail et al. [26] discussed bio-convective thermal radiative nanofluid flow on a stretchy sheet with Darcy-Forchheimer model effects. In the canonical configuration, the surface stretches linearly from a slit, with the velocity proportional to the distance from the origin, generating a boundary layer of constant thickness despite the accelerating surface. This seemingly simple configuration yields elegant analytical solutions for Newtonian fluids, revealing that the stream function varies exponentially with the similarity variable, and that the shear stress at the wall remains constant along the stretching length. This field finds extensive applications in manufacturing processes such as polymer extrusion, metal forming, glass fiber drawing, and continuous casting. Waqas et al. [27] studied Cattaneo-Christov thermal flux model for fluid flow on a stretchable surface by varying thermal conductance. Khan et al. [28] studied modified thermally radiative and convective fluid flow on an elongating cylindrical surface with Cattaneo-Christov fluxes. Das et al. [29] examined thermal diffusion augmentation for nanofluid flow on an elongating sheet with effects of critical share rate. Alqarni et al. [30] studied the dynamics of dissipative nanofluid fluid flow on variable stretchy surface with spinning effects. Real industrial processes introduce considerable complexity through non-linear stretching rates, suction or injection at the surface, magnetic field interactions, and non-Newtonian rheology. When the fluid exhibits shear-thinning or viscoelastic behavior, the momentum boundary layer responds dramatically, with apparent viscosity variations altering velocity profiles, skin friction coefficients, and the entrainment rate of ambient fluid. Hayat et al. [31] studied numerically the thermal transference and radiative effects on stagnant point nanofluid flow on a stretchable cylindrical surface. The thermal aspects prove equally significant, as the stretching surface often emerges at elevated temperatures requiring controlled cooling to achieve desired material properties. Temperature-dependent viscosity, variable thermal conductivity, and sophisticated heat flux models all influence the thermal boundary layer development, determining the final product quality. Li et al. [32] discussed experimentally ferrofluid flow in a stretchable micro-pimps. Researchers have extended the stretching surface concept to numerous configurations including exponential stretching, porous surfaces, unsteady stretching, and multiple stretching sheets in parallel or orthogonal arrangements.

The incorporation of porous media into fluid flow analysis represents a critical advancement in understanding transport phenomena through natural and engineered materials, with the Darcy-Forchheimer model emerging as the most practically relevant framework for moderate to high flow rate conditions. While the classical Darcy law [33] establishes a linear relationship between pressure gradient and velocity, valid for creeping flows through porous matrices, it proves inadequate when inertial effects become significant at higher Reynolds numbers. The Darcy-Forchheimer extension [34] addresses this limitation by introducing a quadratic velocity term that accounts for form drag and inertial losses caused by pore-scale obstacles and tortuous flow paths. The mathematical formulation thus combines viscous resistance, represented by the Darcy term, with inertial resistance, represented by the Forchheimer term, providing a more complete description of momentum transfer through porous structures ranging from packed bed reactors and geothermal reservoirs to biological tissues and filtration media. Islam et al. [35] examined time-dependent mixed convective nanofluid flow with heat sink/source on a permeable medium. Ali et al. [36] studied numerically the Darcy-Forchheimer nanofluid flow on a curved surface. When coupled with fluid motion driven by external pressure gradients, stretching boundaries, or buoyancy forces, the Darcy-Forchheimer model yields fascinating interactions between porous resistance and convective transport. The non-linear Forchheimer term fundamentally alters boundary layer development, velocity profiles, and shear stress distributions compared to purely Darcy or clear fluid flows. Engineers designing thermal insulation systems, electronic cooling devices, catalytic converters, and enhanced oil recovery operations rely on Darcy-Forchheimer analyses to predict pressure drops, flow distributions, and heat transfer rates with greater accuracy than simpler models permit [37–39]. In stretching surface problems, for instance, porous resistance opposes the fluid motion induced by the moving boundary, creating thicker momentum boundary layers and reducing entrainment of ambient fluid. The inertial effects become particularly pronounced near the solid boundary where velocities are highest, while the linear Darcy resistance dominates in regions of lower velocity. Alkasasbeh [40] discussed the modeling for magnetized nanofluid flow with effects of Darcy-Forchheimer model on a surface. The Darcy-Forchheimer framework stands as an indispensable tool for modern porous media research, bridging the gap between idealized low-velocity models and the demanding realities of industrial and environmental flows.

### Novelty

A review of the existing literature reveals a clear research gap: no study has yet investigated the Darcy-Forchheimer bioconvective flow of a Ree-Eyring fluid with thermal relaxation on a stretching surface using a neural network approach subject to effects of inclined magnetic field. The present work addresses this gap by examining this complex flow phenomena. It incorporates the Cattaneo-Christov thermal flux model, binary chemical reaction, inclined magnetic field effects, as well as the influences of Brownian motion and thermophoresis. Furthermore, the flow is subjected to Darcy-Forchheimer and dissipative effects on the permeable stretchable sheet. The main equations have initially evaluated numerically through bvp4c approach in dimensionless form. The dataset generated through the bvp4c numerical scheme is subsequently employed to implement the artificial neural network (ANN) methodology.

## Problem formulation

**Fig. 1:**
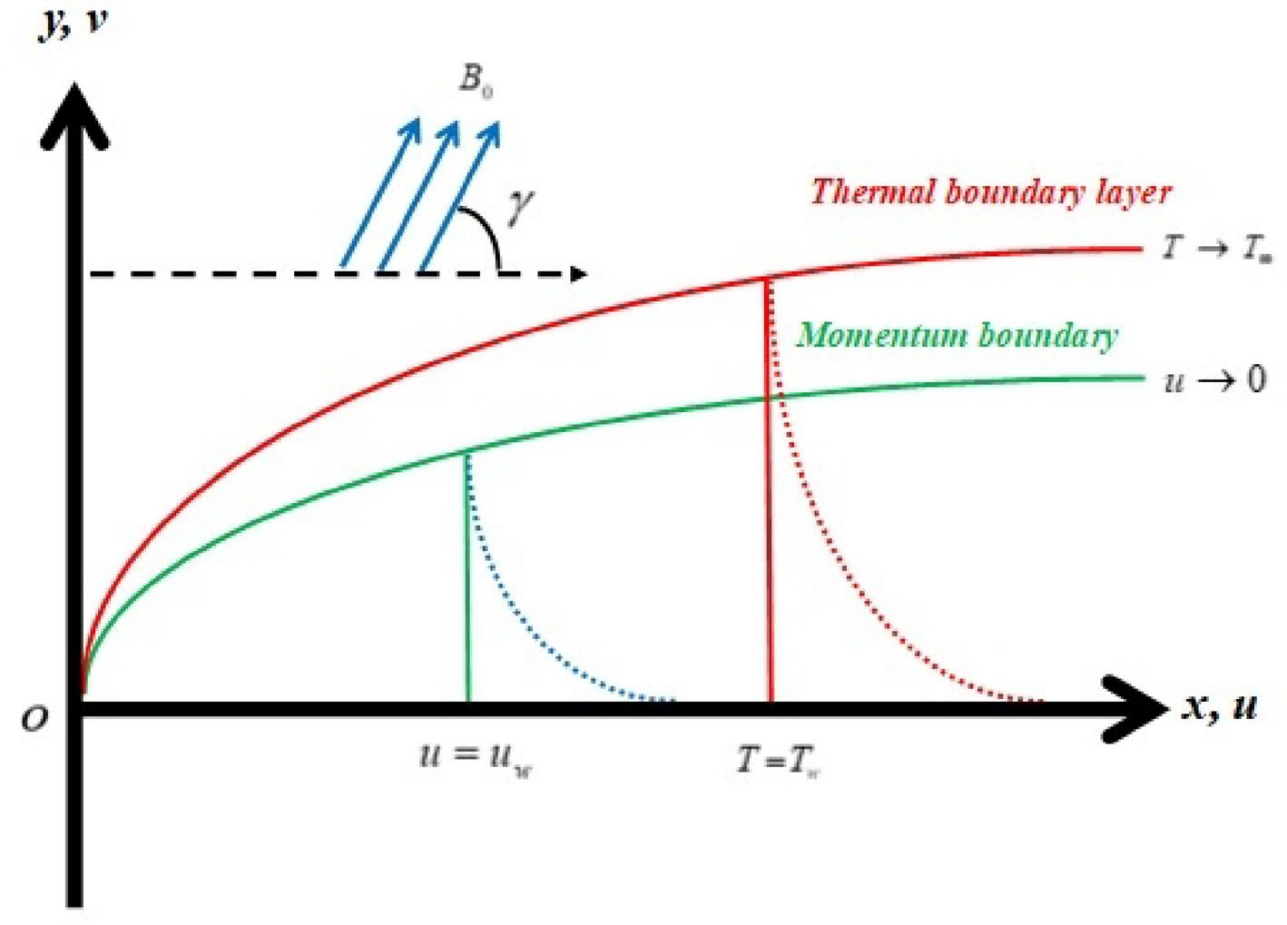
Graphical view of flow problem

Take an incompressible magnetized Ree-Eyring nanofluid flow on an elongating sheet with Darcy-Forchheimer medium effects. The flow is affected by magnetic field of strength ${B_0}$ in inclined direction to fluid motion as portrayed in Fig. 1. The diffusion of temperature is controlled through use of Cattaneo-Christov thermal flux model which is mathematically incorporated in energy equation along with impacts of thermal radiations and heat sink/source. The velocity components are $u,\,\,v$ that are taken along *x* and *y*- axes. The sheet is elongated along *x*-axis with elongating velocity ${u_w}\left( x \right)=px$ with *p>0* as constant. Moreover, the surface and free stream temperatures are kept as ${T_w}$ and ${T_\infty }$.

The Ree-Eyring nanofluid model in terms of stress tensor is mathematically described as [41–43]:

1 $${\tau _{ij}}=\left( {\frac{1}{{\beta A}}{{\sinh }^{ - 1}}\left( {\frac{A}{\varepsilon }} \right)+\mu } \right){B_R}$$

In Eq. 1$A=\sqrt {0.5\,Tr\,{{\left( {{B_R}} \right)}^2}}$, by implementing the stated assumptions we have the leading equations as:

2 $$\frac{{\partial u}}{{\partial x}}+\frac{{\partial v}}{{\partial y}}=0$$

3 $$u\frac{{\partial u}}{{\partial x}}+v\frac{{\partial u}}{{\partial y}}=\frac{1}{\rho }\left( {\frac{1}{{{\beta _1}E}}+\mu } \right)\left( {2\frac{{{\partial ^2}u}}{{\partial {x^2}}}+\frac{{{\partial ^2}u}}{{\partial {y^2}}}+\frac{{{\partial ^2}v}}{{\partial x\partial y}}} \right) - \frac{{\sigma B_{0}^{2}}}{\rho }\cos \left( \gamma \right)u - \frac{\mu }{{\rho {k_1}}}u - \frac{{{c_b}}}{{\sqrt {{k_1}} }}{u^2}$$

4 $$\begin{gathered} u\frac{{\partial T}}{{\partial x}}+v\frac{{\partial T}}{{\partial y}}=\alpha \frac{{{\partial ^2}T}}{{\partial {y^2}}}+\frac{1}{{\rho {C_P}}}\left( {\frac{1}{{{\beta _1}E}}+\mu } \right){\left( {\frac{{\partial u}}{{\partial y}}} \right)^2}\,\,\,\, - \frac{1}{{\rho {C_P}}}\frac{{16{\sigma ^*}}}{{3{k^*}}}{T_\infty }^{3}\frac{{{\partial ^2}T}}{{\partial {y^2}}}+\frac{{{Q_o}}}{{\rho {C_P}}}(T - {T_\infty }) \hfill \\ +\frac{{\sigma B_{0}^{2}}}{{\rho {C_P}}}\cos \left( \gamma \right){u^2}+\frac{\mu }{{{k_1}\rho {C_P}}}{u^2} - \lambda \left[ \begin{gathered} u\frac{{\partial u}}{{\partial x}}\frac{{\partial T}}{{\partial x}}+v\frac{{\partial v}}{{\partial y}}\frac{{\partial T}}{{\partial y}}+u\frac{{\partial v}}{{\partial x}}\frac{{\partial T}}{{\partial y}}+ \hfill \\ v\frac{{\partial u}}{{\partial y}}\frac{{\partial T}}{{\partial x}}+2uv\frac{{{\partial ^2}T}}{{\partial x\partial y}}+{u^2}\frac{{{\partial ^2}T}}{{\partial {x^2}}}+{v^2}\frac{{{\partial ^2}T}}{{\partial {y^2}}} \hfill \\ \end{gathered} \right] \hfill \\ \end{gathered} $$

The physical conditions at boundaries are:

5 $$\left. \begin{gathered} u={u_w},\quad v={v_w},\quad T={T_w},\quad \quad \quad {\text{at }}y=0 \hfill \\ u \to 0,\quad T \to {T_\infty },\quad \,\,\,\,\,\,\,\,\,\,\,\,\,\,\,\,\,\,\quad \quad {\text{as }}y \to \infty \hfill \\ \end{gathered} \right\}$$

The Ree-Eyring model was chosen because it is derived from kinetic theory and is particularly suitable for describing the behavior of non-Newtonian fluids exhibiting shear-thinning characteristics while maintaining a finite viscosity at both low and high shear rates. Unlike the Casson model, which is primarily intended for yield-stress fluids such as blood. The Ree-Eyring model does not require the presence of a yield stress and is therefore more appropriate for the class of fluids considered in the present study. Furthermore, although the Carreau model effectively captures shear-rate-dependent viscosity, the Ree-Eyring model offers a mathematically tractable constitutive relation that accurately represents non-linear stress-strain behavior under moderate and high shear conditions commonly encountered in stretching-surface and rotating-disk flows. Since the present investigation involves stagnation-point flow over a stretching/rotating surface where significant shear effects arise, the Ree-Eyring model provides a realistic balance between physical accuracy.

Using the following transformable variables for non-dimensionalization

6 $$\left. {u=pxF^{\prime}(\eta ),\quad v= - \sqrt {p\nu } {\mkern 1mu} F(\eta ),\quad \eta =\sqrt {\frac{p}{\nu }} {\mkern 1mu} y,\,\,\,\,\,\Theta (\eta )({T_w} - {T_\infty })=T - {T_\infty }} \right\}.$$

Using Eq. 6 in Eqs. 2–5 we have:

7 $$(1+We)F^{\prime\prime\prime} - (M\cos \left( \gamma \right)+K)F^{\prime}+FF^{\prime\prime} - Fr{(F^{\prime})^2} - {(F^{\prime})^2}=0$$

8 $$\left( {\left( {1+\frac{4}{3}Rd} \right) - Pr\delta {F^2}} \right)\Theta ^{\prime\prime}+\Pr \left[ \begin{gathered} F\Theta ^{\prime} - \delta FF^{\prime}\Theta ^{\prime}+MEc{(F^{\prime})^2}\,\cos \left( \gamma \right)+(1+We)Ec{(F^{\prime\prime})^2} \hfill \\ \,\,\,\,\,\,\,\,\,\,\,\,\,\,\,\,\,\,\,\,\,\,\,\,\,\,\,\,\,\,\,\,\,\,\,\,\,\,\,\,\,\,\,\,\,\,\,\,\,\,\,\,\,\,\,\,\,\,\,\,\,\,\,\,\,\,\,\,\,\,\,\,\,\,\,+Q\Theta +KEc{(F^{\prime})^2} \hfill \\ \end{gathered} \right]=0$$

The physical conditions in dimensionless form are:

9 $$F(0)=S,\quad F^{\prime}(0)=1,\quad \Theta (0)=1,\,\,\,\,\,F^{\prime}(\infty )=0,\quad \Theta (\infty )=0.$$

In above equations we have $We=\frac{1}{{\mu E{\beta _1}}}=$ Weissenberg number, $K=\frac{v}{{{k_1}p}}=$ porosity factor, $Fr=\frac{{{c_b}}}{{\sqrt {{k_1}} }}=$ Darcy Forchheimer factor, $M=\frac{{\sigma B_{0}^{2}}}{{{\rho _f}p}}=$ magnetic parameter, $Rd=\frac{{4{\sigma ^*}T_{\infty }^{3}}}{{k{k^*}}}=$ radiation factor, $Pr=\frac{v}{\alpha }=$ Prandtl number, $\delta =p\lambda =$ thermal relaxation time parameter, $Q=\frac{{{Q_0}}}{{a{{\left( {\rho {C_p}} \right)}_f}}}=$ heat source factor and $Ec=\frac{{u_{w}^{2}}}{{{C_p}({T_w} - {T_\infty })}}=$ Eckert number.

### Important quantities of engineering interest

The quantities of engineering interest in current study are skin friction and Nusselt number which are illustrated in dimensionless form as:

10 $$ \sqrt {Re_{x} } C_{{f_{x} }} = 2(1 + We)F^{{\prime \prime }} \left( 0 \right) = {\mathrm{skin}}\;{\mathrm{friction}},\,\frac{{Nu_{x} }}{{\sqrt {Re_{x} } }} = - \left( {1 + \frac{4}{3}R} \right)\Theta ^{\prime } \left( 0 \right) = {\mathrm{Nusselt}}\;{\mathrm{number}} $$

## Methodology

To solve the coupled system of ODEs presented in Eqs. 7 and 8, in conjunction with the associated boundary conditions given in Eq. 9, we employ the bvp4c numerical approach. This solver, implemented in MATLAB, is specifically designed to handle boundary value problems with robust accuracy. The bvp4c method is particularly well-suited for this analysis due to its strong convergence characteristics, enabling it to efficiently resolve highly nonlinear equations. By discretizing the governing equations and iteratively adjusting the solution to satisfy boundary conditions, this technique ensures reliable and precise numerical results for complex fluid flow problems. In this method the higher order ODEs are first need to convert into 1st order ODE as follows.

So let us assumed that

11 $$F={\varpi _1},\,\,\,F^{\prime}={\varpi _2},\,\,\,F^{\prime\prime}={\varpi _3},\,\,\,\Theta ={\varpi _4},\,\,\,\Theta ^{\prime}={\varpi _5}.$$

As a result of Eq. 11, we get:

12 $$(1+We){\varpi ^{\prime}_3} - (M\cos \left( \gamma \right)+K){\varpi _2}+{\varpi _1}{\varpi _3} - Fr{({\varpi _2})^2} - {({\varpi _2})^2}=0,$$

13 $$\left( {\left( {1+\frac{4}{3}Rd} \right) - Pr\delta {\varpi _1}^{2}} \right){\varpi ^{\prime}_5}+Pr\left[ \begin{gathered} {\varpi _1}{\varpi _5} - \delta {\varpi _1}{\varpi _2}{\varpi _4}+MEc{({\varpi _2})^2}\,\cos \left( \gamma \right)+(1+We)Ec{({\varpi _3})^2} \hfill \\ \,\,\,\,\,\,\,\,\,\,\,\,\,\,\,\,\,\,\,\,\,\,\,\,\,\,\,\,\,+Q{\varpi _4}+Ec{({\varpi _2})^2}K \hfill \\ \end{gathered} \right]=0.$$

The transform conditions in dimensionless form are:

14 $${\varpi _1}(0)=S,\quad {\varpi _2}(0)=1,\quad {\varpi _4}(0)=1,\,\,\,\,\,{\varpi _2}(\infty )=0,\quad {\varpi _4}(\infty )=0.$$

**Fig. 2:**
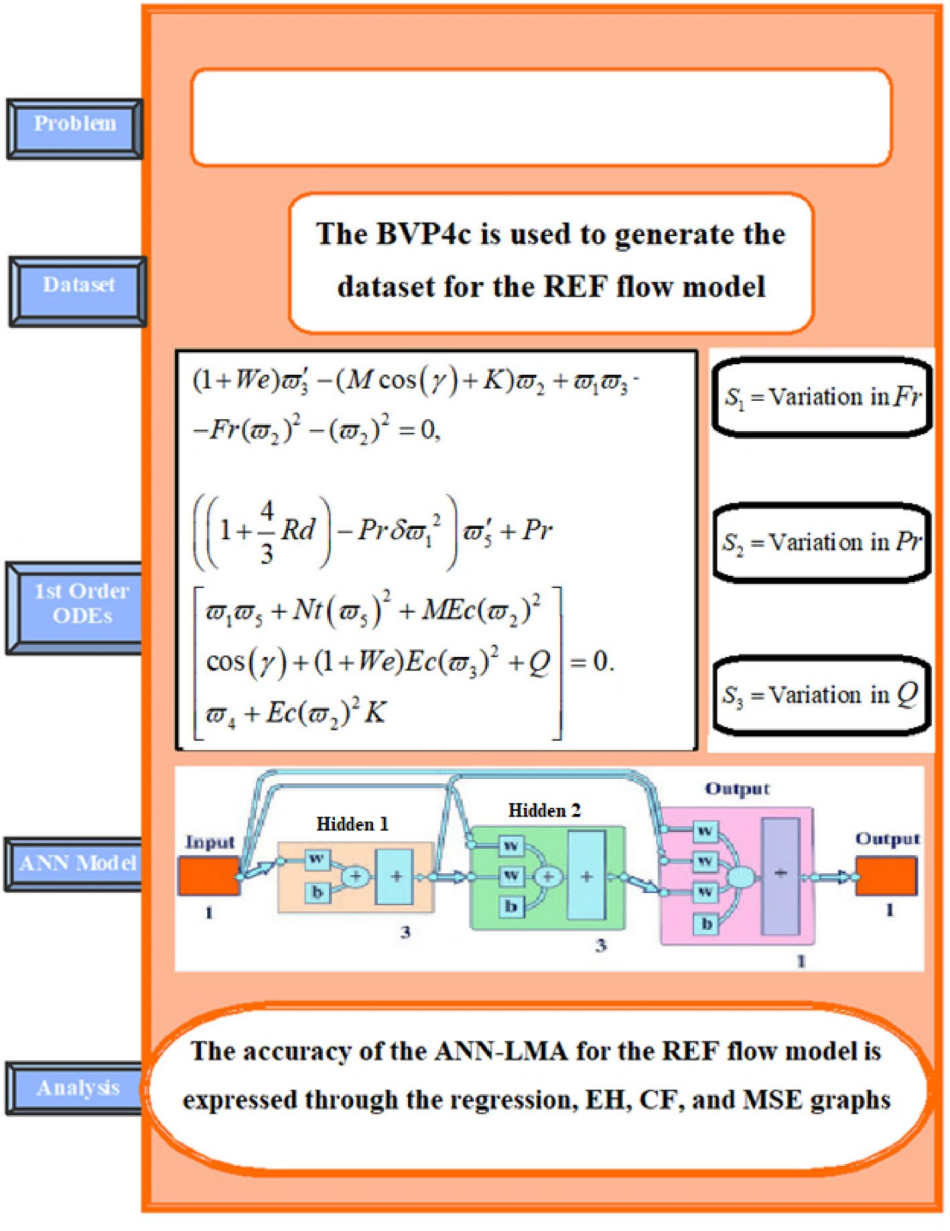
ANN-LMA layout for modeled problem

To train the ANN model, a dataset consisting of 140 data points was generated using the bvp4c solver, partitioned into a precise split ratio of 70% for training, 15% for validation, and 15% for testing. For the ANN-LMA, the detailed layout is shown in Fig. 2. This figure illustrates the LMS-NNA architecture, highlighting its optimization using mean squared error (MSE). The diagram presents the structure and data flow across the input, hidden, and output layers, enabling a comprehensive evaluation of the network design, parameter tuning, and predictive performance.

## Results and discussion

This work investigates the magnetohydrodynamic flow of a dissipative Ree-Eyring fluid over a permeable stretching surface, subjected to an inclined magnetic field relative to the direction of fluid motion. Thermal transport is analyzed using the Cattaneo-Christov heat flux model, which accounts for thermal relaxation effects absent in the classical Fourier formulation. The flow regime further incorporates the influence of a Darcy-Forchheimer porous medium, introducing both linear and quadratic drag contributions to the momentum equation. The main equations have initially evaluated numerically through bvp4c approach in dimensionless form. The dataset generated through the bvp4c numerical scheme is subsequently employed to implement the artificial neural network (ANN) methodology.

### Analysis of ANN graphs

**Fig. 3:**
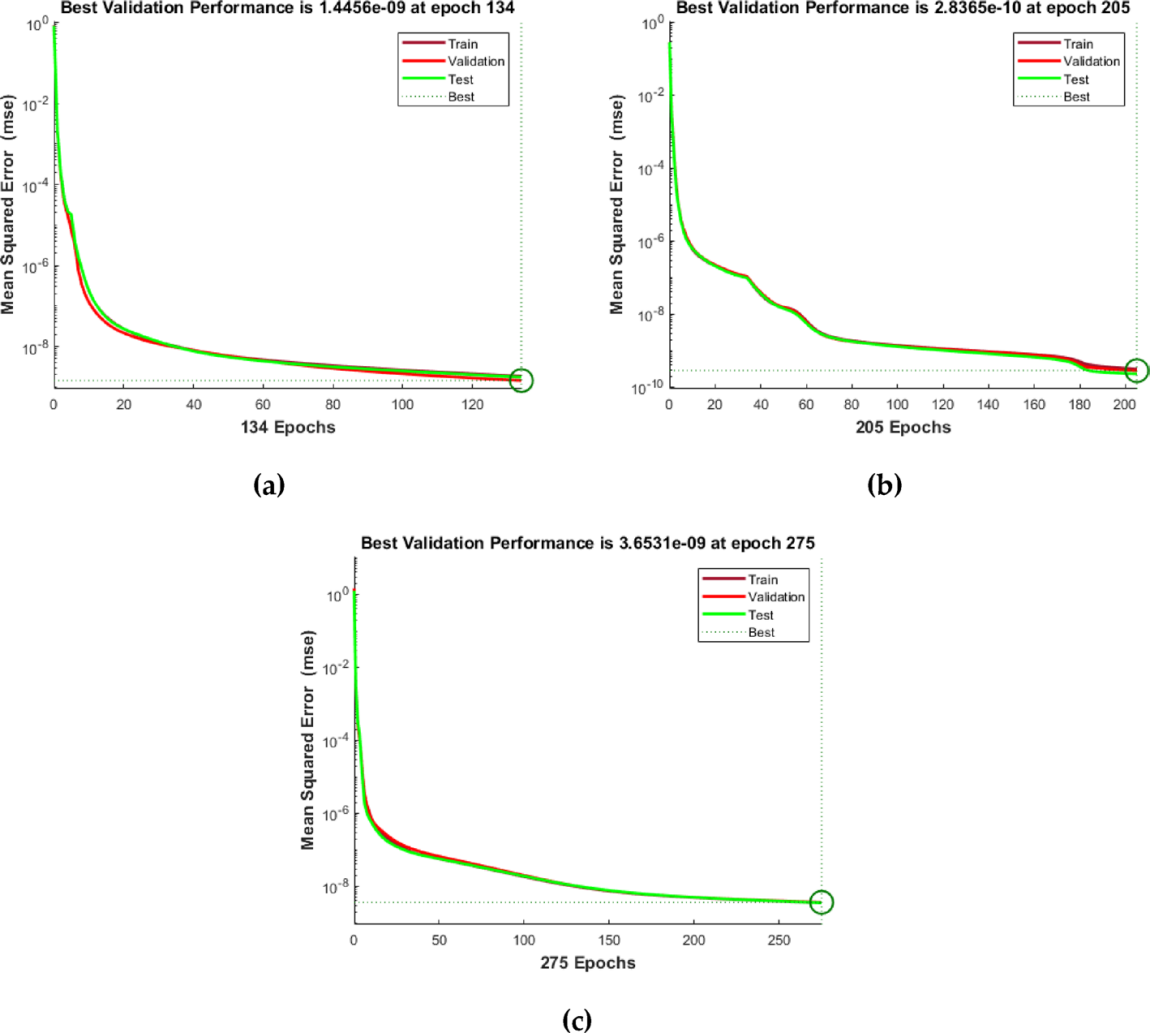
**a-c** MSE for ANN design (Scenarios 1–3)

**Fig. 4:**
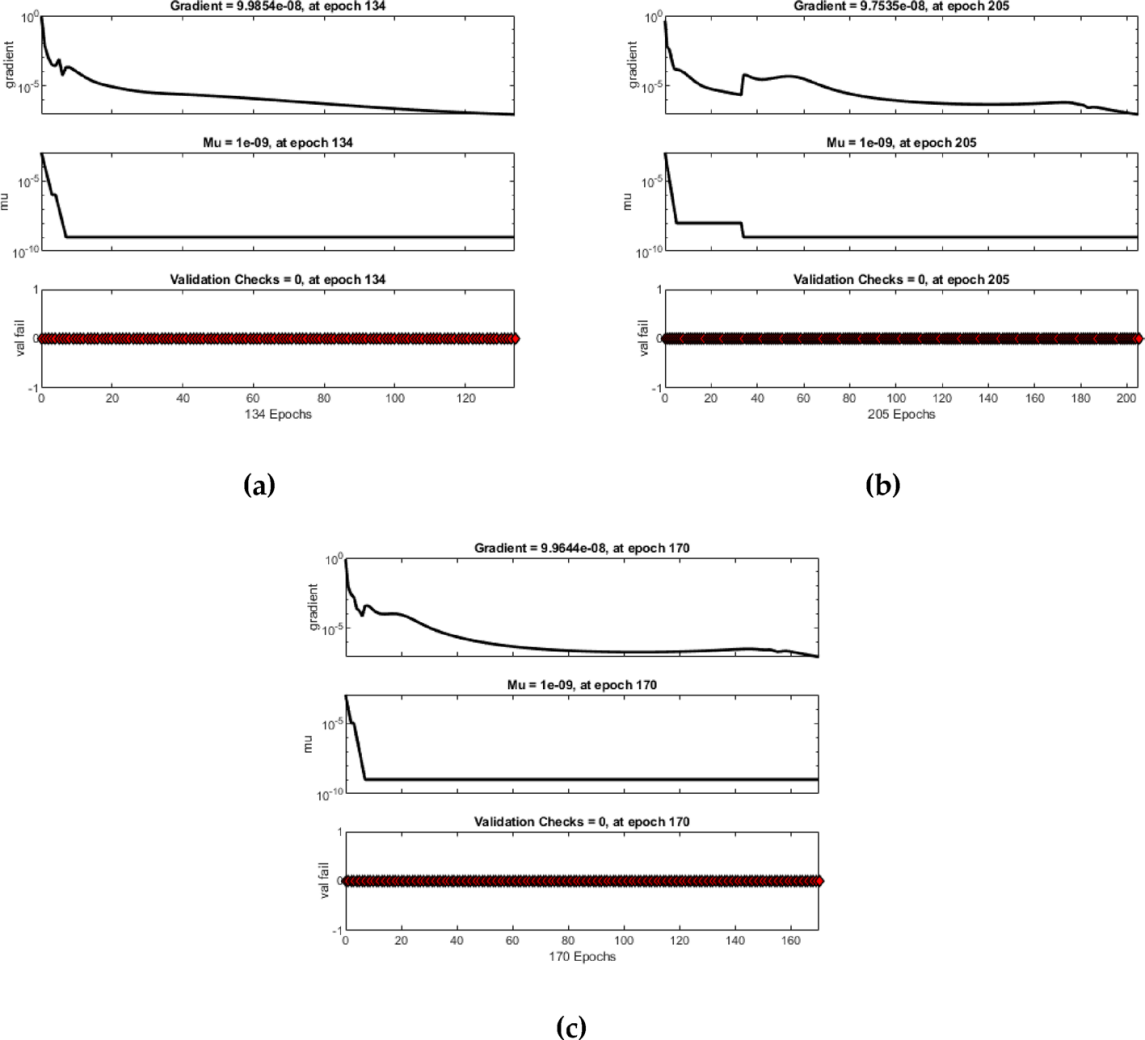
**a-c** Transition state for ANN design (Scenarios 1–3)

**Fig. 5:**
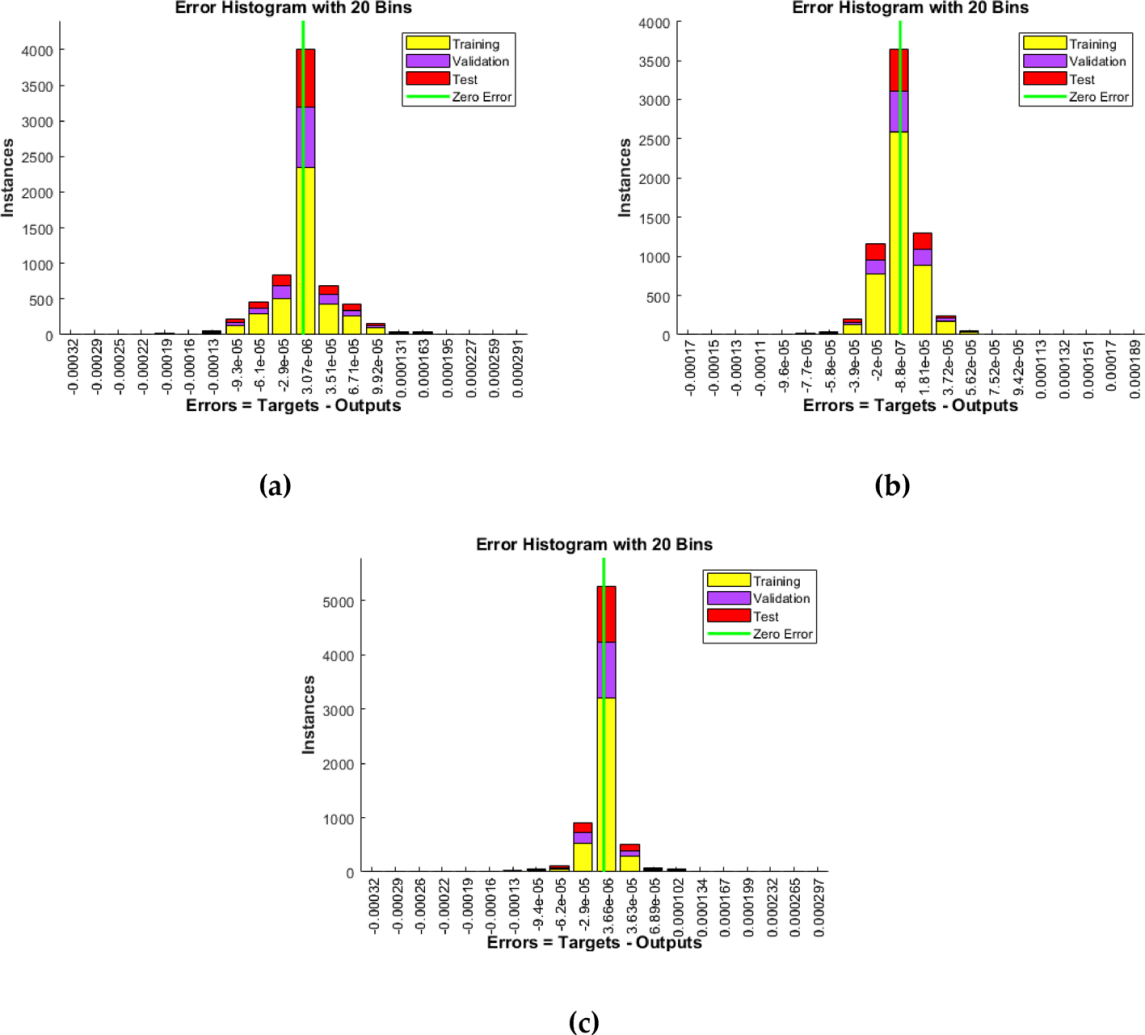
**a-c** Error histogram for ANN design (Scenarios 1–3)

**Fig. 6:**
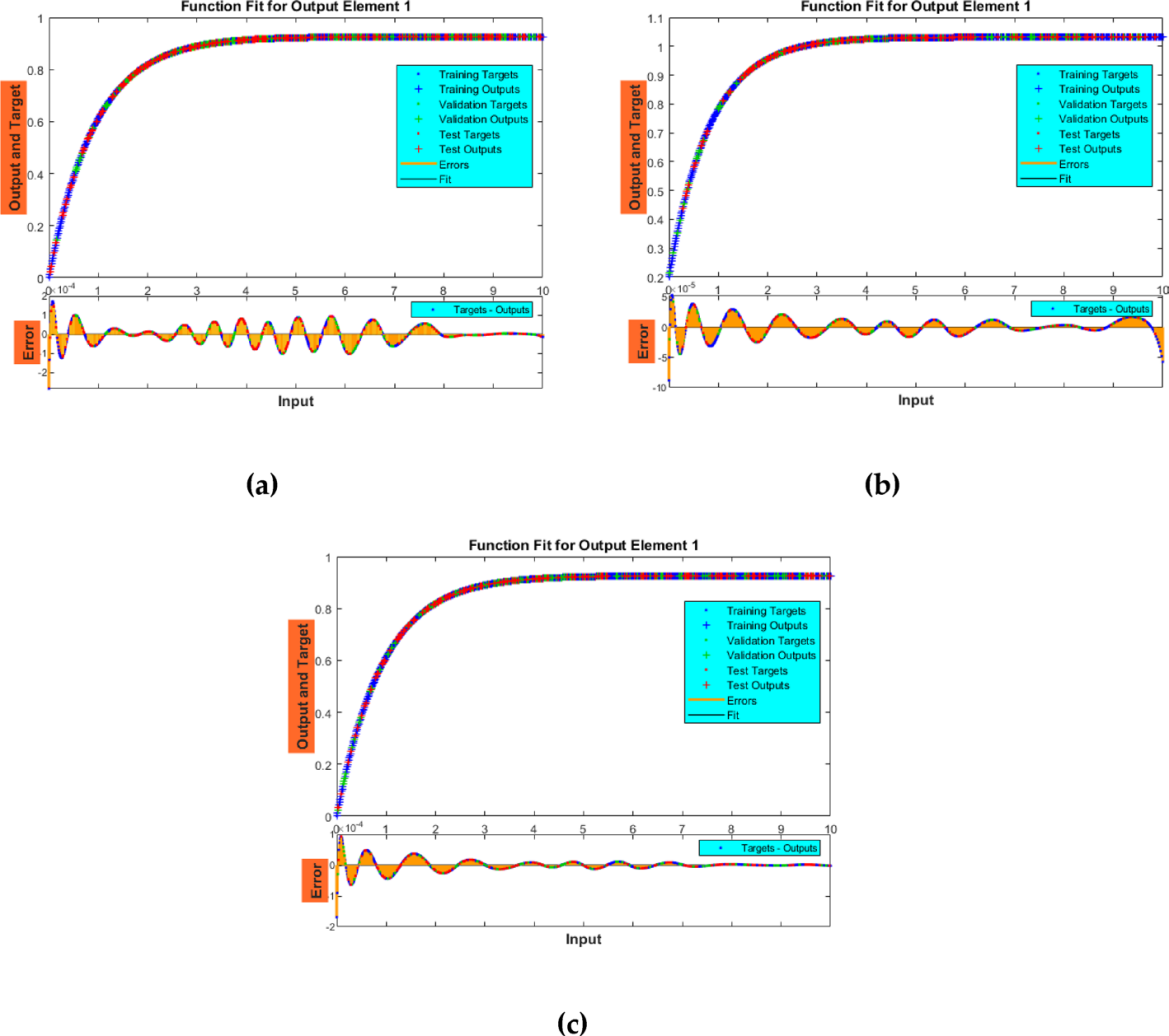
**a-c** Curve fitting for ANN design (Scenarios 1–3)

**Fig. 7:**
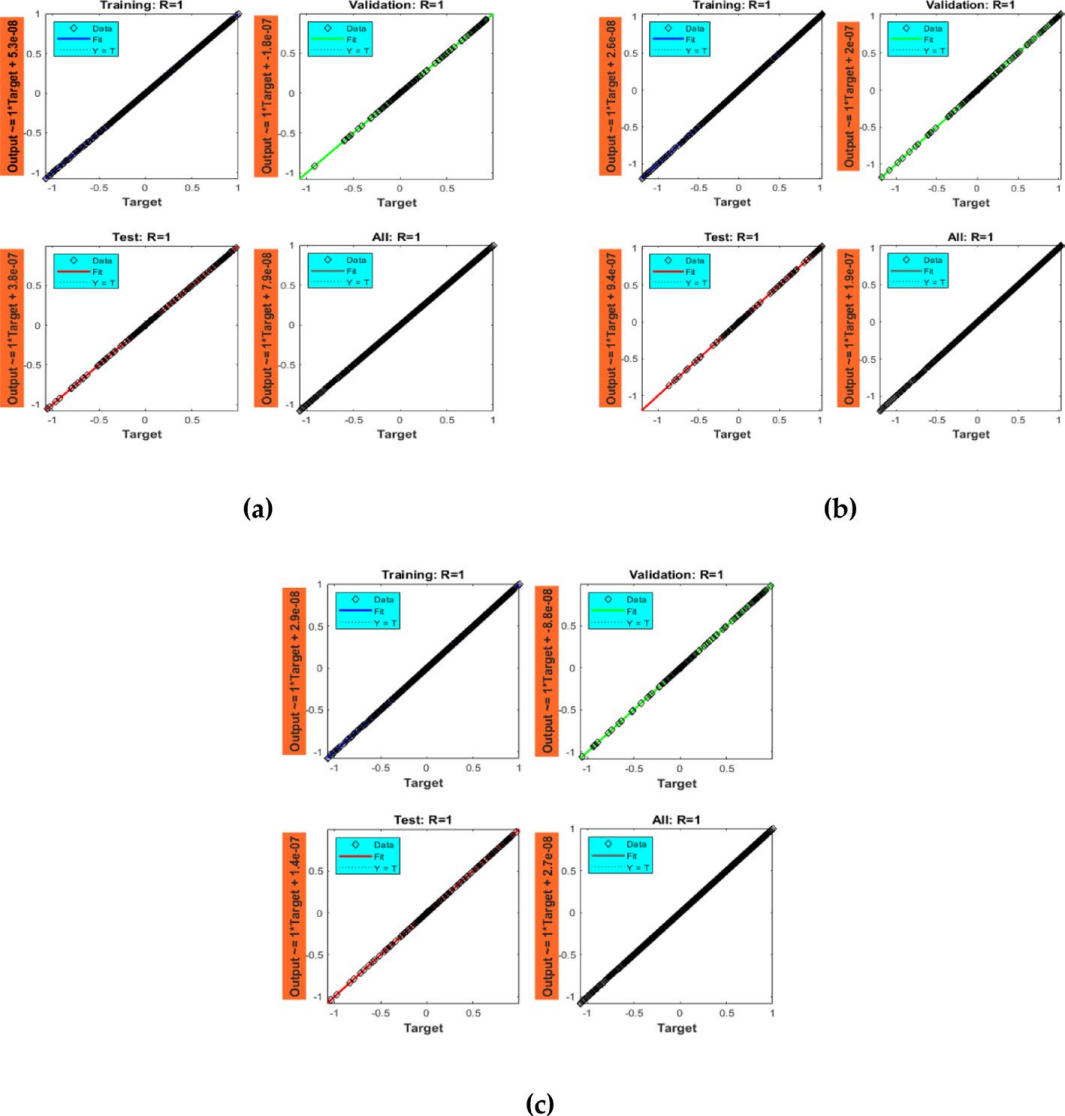
**a-c** Regression analysis for ANN design (Scenarios 1–3)

The Levenberg-Marquardt scheme combined with a neural network algorithm (LMS-NNA) is employed to guide the learning process within a multilayer perceptron (MLP), a widely used ANN model. In this approach, the network learns and stores information by adjusting the weights assigned to connections between the input, hidden, and output neurons. During training, the LMS-NNA optimizes these weights to minimize error, enabling the MLP to detect patterns, improve prediction accuracy, and generalize effectively to new data. This approach is particularly effective for solving complex, nonlinear problems. The architecture of the network consists of a multi-layer Cascade Forward neural network made up of two successive hidden layers of 3 neurons respectively. It was selected due to the various combinations evaluated, which have the lowest Mean Squared Error (MSE) and do not cause overfitting or excessive computational load. Continuous weight adjustment further enhances the model’s generalization capability, ensuring reliable performance on unseen data. Overall, the integration of the MLP architecture with the LMS-NNA algorithm provides a robust framework for developing advanced neural models capable of addressing complex, nonlinear tasks across various applications. Figures 3, 4, 5, 6 and 7 present the ANN-based analysis for the design problem. Specifically, Fig. 3(a-c) show the convergence of MSE during training across all the scenarios. MSE, defined as the average squared difference between predicted and target values, serves as the primary performance metric. These plots include results from the training, testing, and validation phases, with optimal convergence achieved at epochs 134, 205 and 275 for the respective cases. Figure 4(a-c) depict the transitional behavior of the ANN model, capturing the iterative improvement in predictions. These graphs illustrate the model’s progression from an initial approximation to a refined solution, as it increasingly captures the coupled effects of velocity, temperature, and concentration fields governing momentum, heat, and mass transfer. Figure 5(a-c) present error histograms for all the three scenarios, offering insight into model accuracy and convergence. These plots display the distribution of prediction errors, confirm solution stability over iterations, and demonstrate close agreement between predicted and expected results. Figure 6(a-c) focus on the fitness evaluation of the error analysis (EA), showing how solution quality improves across different stages. The plots include error evaluations for input values ranging from 0 to 5 in increments of 0.5, providing a detailed view of model optimization. Finally, Fig. 7(a-c) display regression performance for all the three scenarios. The correlation coefficients (R) for the training, testing, and validation datasets are consistently close to 1, indicating strong agreement between predicted and actual values and confirming the high accuracy and reliability of the ANN model.

### Analysis of fluid flow graphs

The following sections provide a theoretical investigation into the effects of various governing parameters. This analysis is supported by graphical illustrations depicting velocity and temperature distributions in fluid flow through a porous medium across a stretching surface. The objective is to clarify the complex physical behavior involved in this system and to better understand how different parameters influence flow characteristics. By combining theoretical interpretation with visual representation, the study offers deeper insight into the coupled mechanisms controlling momentum and heat transfer within the medium. These findings help to explain variations in flow patterns and thermal profiles, contributing to a more comprehensive understanding of the underlying physics.

### Velocity profiles

**Fig. 8:**
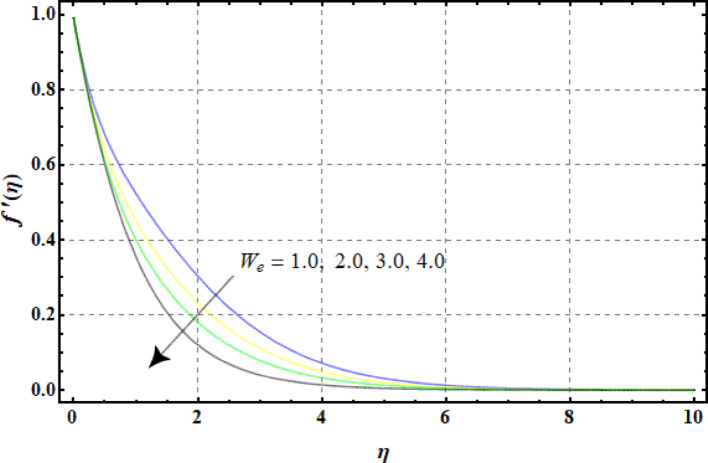
Effect of ${W_e}$ on $f^{\prime}\left( \eta \right)$

**Fig. 9:**
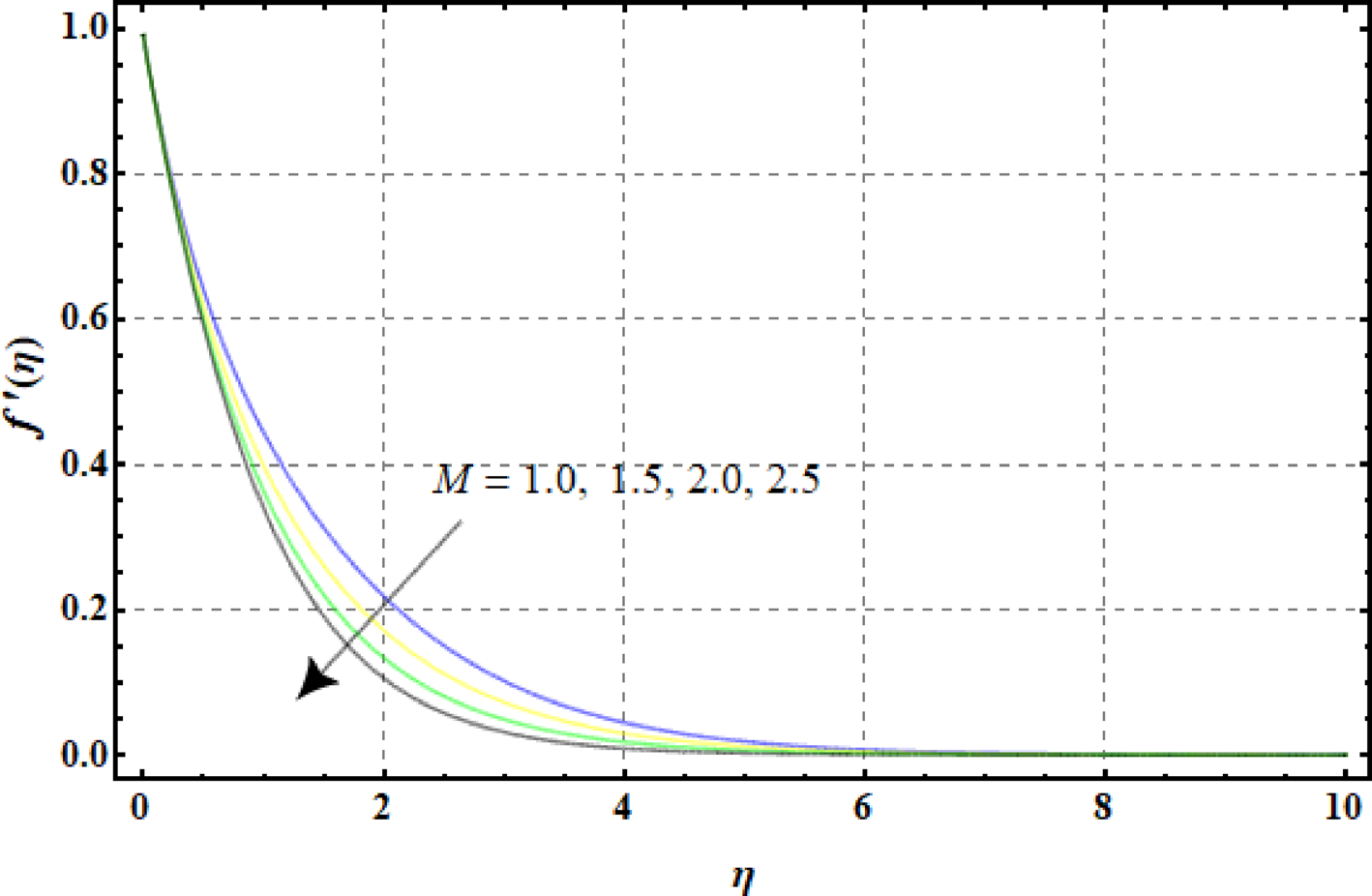
Effect of *M* on $f^{\prime}\left( \eta \right)$

**Fig. 10:**
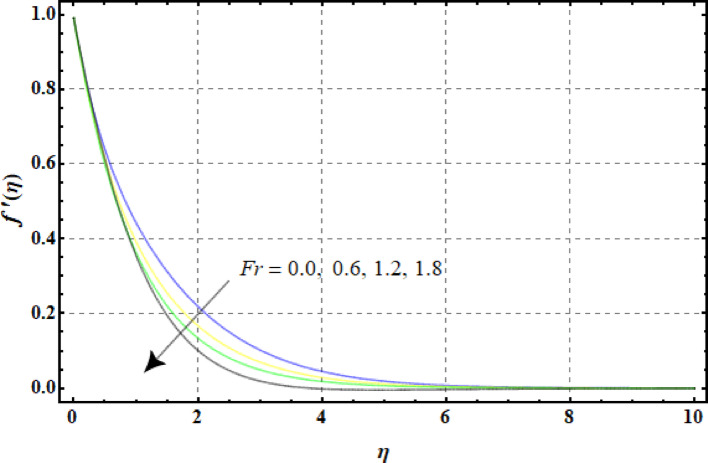
Effect of *Fr*on $f^{\prime}\left( \eta \right)$

**Fig. 11:**
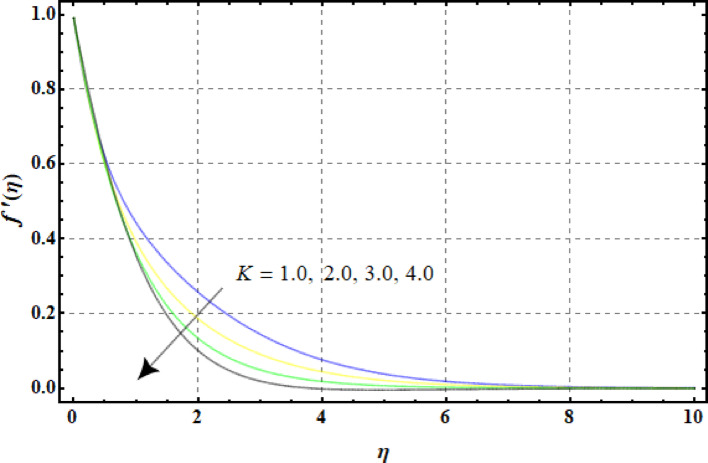
Effect of *K* on $f^{\prime}\left( \eta \right)$

The influences of numerous parameters on velocity profiles $\left\{ {f^{\prime}\left( \eta \right)} \right\}$ are examined in Figs. 8, 9, 10, and 11. The impression of Weissenberg number $\left( {We} \right)$ on $\left\{ {f^{\prime}\left( \eta \right)} \right\}$ is explained in Fig. 8. It is detected from this figure that with growth in $\left( {We} \right)$ there is reduction in $\left\{ {f^{\prime}\left( \eta \right)} \right\}$. Actually, the decline in $\left\{ {f^{\prime}\left( \eta \right)} \right\}$ with increasing $\left( {We} \right)$ in a Ree-Eyring fluid flow across a permeable stretching surface arises from the dominance of elastic behavior over viscous effects. The Weissenberg number characterizes the ratio of elastic to viscous forces, so as $\left( {We} \right)$ rises, the fluid exhibits greater resistance to deformation due to its elastic nature, effectively stiffening the flow. For fluid flow on surface with permeability, this elasticity counteracts the momentum imparted by the stretching motion, reducing the fluid’s ability to be drawn along the surface. Additionally, the permeability introduces localized resistance/suction effects that further diminishes momentum transmission. Consequently, higher $\left( {We} \right)$ leads to suppressed velocity profiles $\left\{ {f^{\prime}\left( \eta \right)} \right\}$, reflecting a transition where elastic stresses increasingly hinder fluid motion relative to viscous shearing. The impression of magnetic parameter $\left( M \right)$ on $\left\{ {f^{\prime}\left( \eta \right)} \right\}$is explained in Fig. 9. It is detected from this figure that with growth in $\left( M \right)$ there is reduction in $\left\{ {f^{\prime}\left( \eta \right)} \right\}$. From a fluid mechanics perspective, the decline in $\left\{ {f^{\prime}\left( \eta \right)} \right\}$ with increasing $\left( M \right)$ in a Ree-Eyring fluid flow across a permeable stretching surface is attributed to the retarding effect of the Lorentz force. The magnetic parameter quantifies the strength of the applied magnetic field relative to inertial forces, so when as it augments, the Lorentz force, which opposes the fluid motion, becomes more noticeable. This force acts in the direction opposite to the flow, effectively generating a resistive drag that impedes momentum transport. In the context of a extending surface with permeability, this magnetic damping is further modulated by the suction or injection effects inherent to the permeable boundary. The combined influence of the Lorentz force and permeability restricts the propagation of velocity from the stretching surface into the fluid, resulting in thinner momentum layers at boundary and suppressed velocity profiles $\left\{ {f^{\prime}\left( \eta \right)} \right\}$. Thus, higher $\left( M \right)$ enhance the electromagnetic braking effect, reducing the fluid’s ability to follow the stretching motion. The impression of Darcy Forchheimer factor $\left( {Fr} \right)$ on $\left\{ {f^{\prime}\left( \eta \right)} \right\}$ is explained in Fig. 10. It is detected from this figure that with growth in $\left( {Fr} \right)$ there is reduction in $\left\{ {f^{\prime}\left( \eta \right)} \right\}$. Physically, the decline in $\left\{ {f^{\prime}\left( \eta \right)} \right\}$ with increasing $\left( {Fr} \right)$ in a Ree-Eyring fluid flow across the permeable surface arises from the enhanced resistance offered by the porous medium. The Darcy-Forchheimer factor accounts for both viscous drag (Darcy term) and inertial drag (Forchheimer term) within the porous structure. As this factor rises, the porous medium exerts a greater opposing force on the fluid motion. On the surface with permeability, this heightened resistance restricts the transmission of momentum from the stretching boundary into the fluid bulk. The Ree-Eyring fluid’s shear-thinning behavior further modulates this interaction, but the dominant effect remains the substantial pressure drop and energy dissipation caused by the porous matrix. Consequently, higher $\left( {Fr} \right)$ leads to suppressed velocity profiles $\left\{ {f^{\prime}\left( \eta \right)} \right\}$, reflecting the increased impedance to flow and reduced momentum penetration depth within the porous medium. The impression of porosity parameter $\left( K \right)$ on $\left\{ {f^{\prime}\left( \eta \right)} \right\}$ is explained in Fig. 11. It is detected from this figure that with growth in $\left( K \right)$ there is decline in $\left\{ {f^{\prime}\left( \eta \right)} \right\}$. As $\left( K \right)$ escalates, the resistance offered by the porous medium becomes stronger, generating an additional drag force that opposes the fluid motion. Consequently, the velocity $\left\{ {f^{\prime}\left( \eta \right)} \right\}$ decreases and the momentum boundary layer becomes thinner due to the reduced penetration of momentum away from the surface. The reduction in boundary-layer thickness is not strictly linear, since the porous-medium resistance enters the momentum equation in a coupled nonlinear manner and interacts with other flow parameters. This nonlinear interaction causes the momentum penetration depth to decrease at a non-uniform rate with increasing $\left( K \right)$. Physically, the decline in $\left\{ {f^{\prime}\left( \eta \right)} \right\}$ with increasing $\left( K \right)$ in a Ree-Eyring fluid flow across the surface is attributed to the heightened resistance offered by the porous medium. The porosity parameter characterizes the permeability of the medium, so as it surges, the porous structure becomes more restrictive to fluid motion, effectively reducing the ease with which the fluid can flow through spaces on sheet’s surface. For fluid flow across the surface with permeability, higher porosity amplifies the drag forces exerted by the solid matrix on the fluid, thereby impeding momentum transfer from the stretching boundary into the fluid bulk. This enhanced flow resistance dissipates kinetic energy more rapidly, resulting in thinner momentum layer at borderline and suppressed velocity profiles. Thus, a rise in $\left( K \right)$ intensifies the obstructive effect of the porous medium, leading to a notable reduction in fluid velocity throughout the flow domain.

### Thermal profiles

**Fig. 12:**
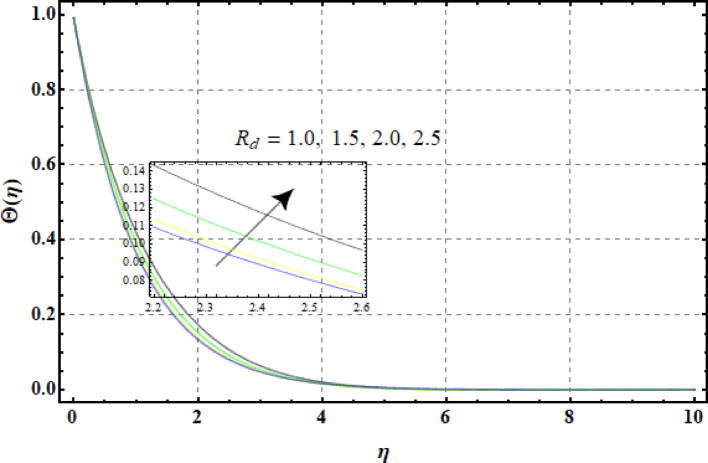
Effect of ${R_d}$ on $\theta \left( \eta \right)$

**Fig. 13:**
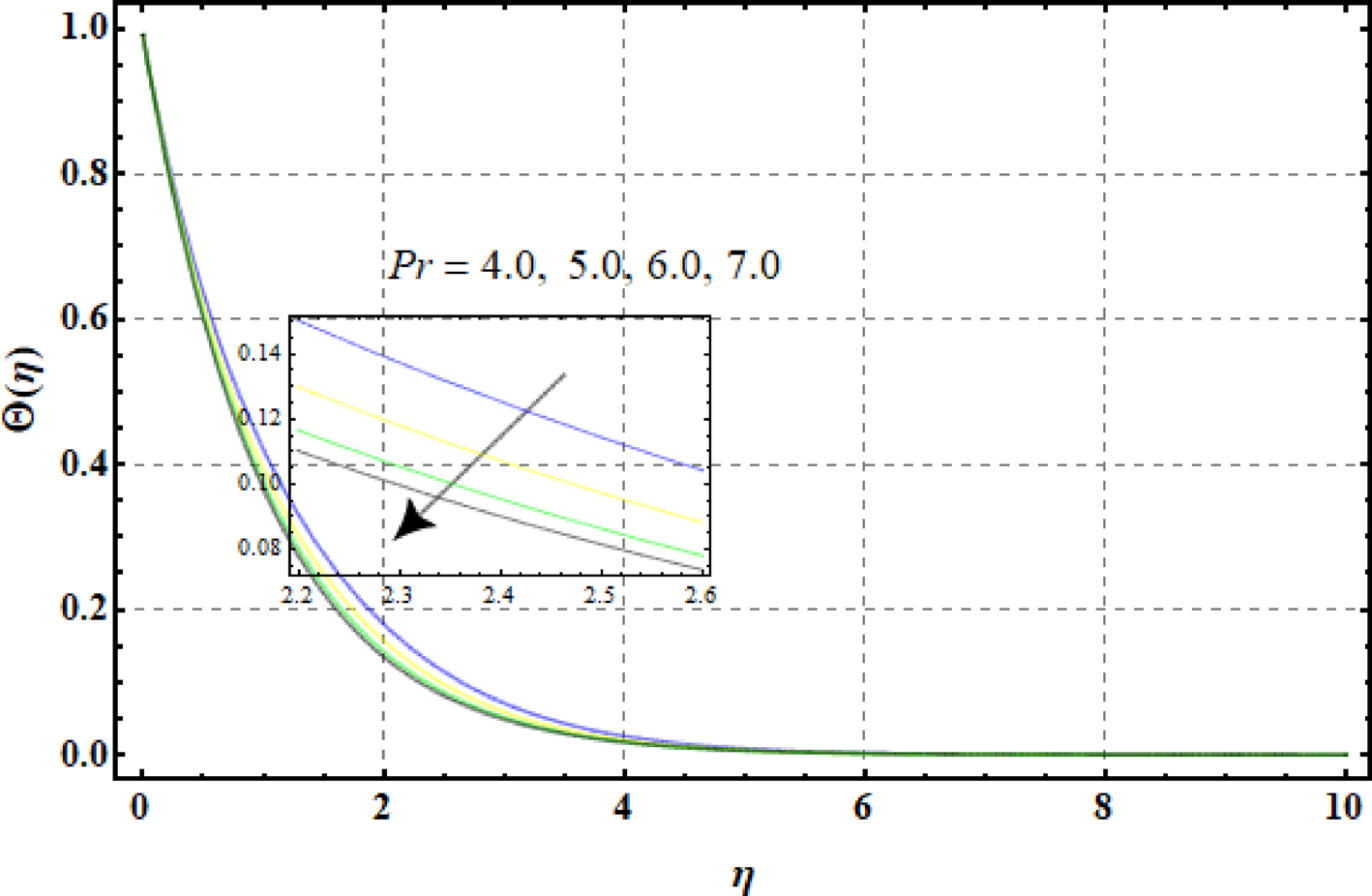
Effect of *\Pr * on $\theta \left( \eta \right)$

**Fig. 14:**
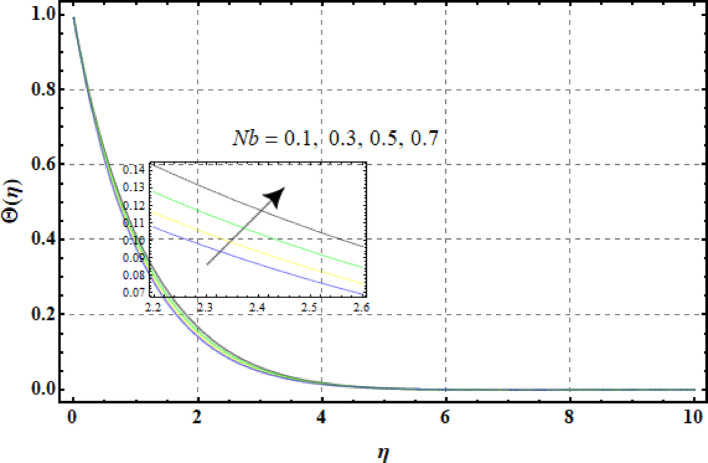
Effect of *Nb* on $\theta \left( \eta \right)$

**Fig. 15:**
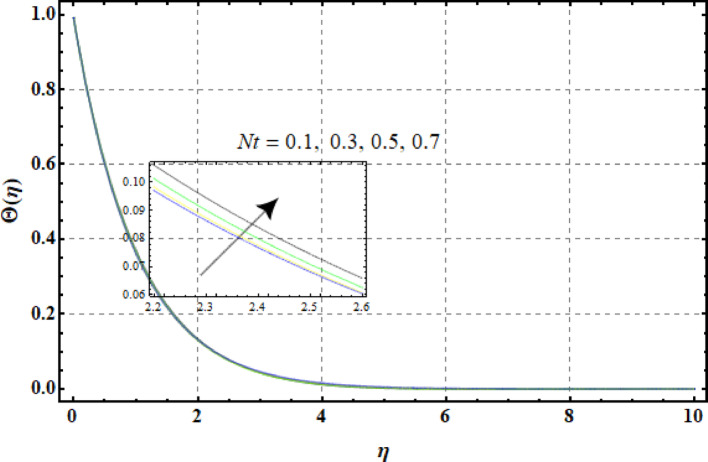
Effect of *Nt* on $\theta \left( \eta \right)$

**Fig. 16:**
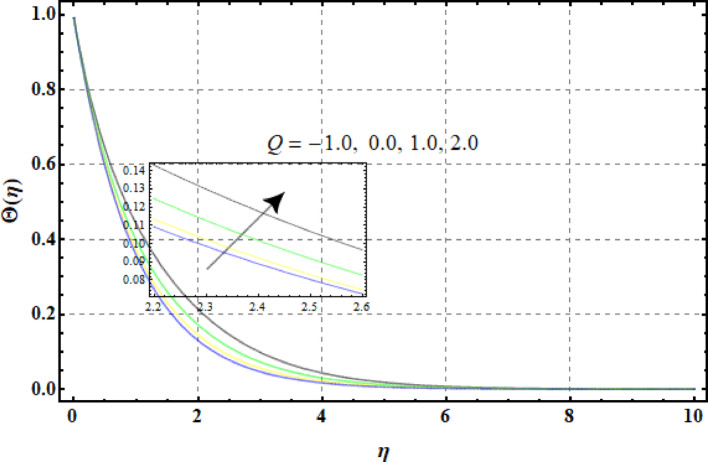
Effect of *Q* on $\theta \left( \eta \right)$

**Fig. 17:**
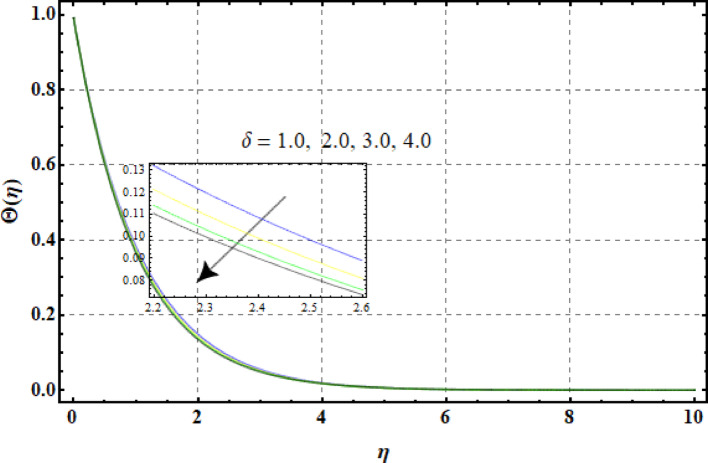
Effect *δ * on $$\theta \left( \eta \right)$$

The influences of numerous parameters on thermal profiles $\left\{ {\theta \left( \eta \right)} \right\}$ are examined in Figs. 12, 13, 14, 15, 16, and 17. The impression of radiation parameter $\left( {Rd} \right)$ on $\left\{ {\theta \left( \eta \right)} \right\}$ is explained in Fig. 12. With augmentation in $\left( {Rd} \right)$ there is surge in $\left\{ {\theta \left( \eta \right)} \right\}$. From a fluid mechanics and heat transfer perspective, the augmentation in $\left\{ {\theta \left( \eta \right)} \right\}$ with increasing $\left( {Rd} \right)$ in a Ree-Eyring fluid flow across the surface is attributed to the enhanced contribution of thermal radiation to the overall energy transfer process. Physically, as $\left( {Rd} \right)$ rises, the medium becomes more optically thick with which the radiative emissivity intensifies, allowing thermal energy to penetrate deeper into the flow domain. For fluid flow across the surface with permeability, this additional radiative energy supplements the conductive heat transfer from the heated boundary, effectively increasing the thermal layer thickness and elevating fluid temperatures. The Ree-Eyring fluid’s shear-dependent viscosity further influences the temperature distribution, but the dominant effect remains the surplus energy supplied by radiation, which enhances thermal diffusion and results in significantly higher temperature profiles $\left\{ {\theta \left( \eta \right)} \right\}$ across the flow field. The impression of Prandtl number $\left( {\Pr } \right)$ on $\left\{ {\theta \left( \eta \right)} \right\}$ is explained in Fig. 13. With augmentation in $\left( {\Pr } \right)$ there is decline in $\left\{ {\theta \left( \eta \right)} \right\}$. A physical interpretation of the decline in $\left\{ {\theta \left( \eta \right)} \right\}$ with increasing $\left( {\Pr } \right)$ is that a higher Prandtl number signifies a fluid where momentum diffuses much more effectively than heat. Consequently, for a given flow, the viscous (momentum) boundary layer thickens rapidly compared to the thermal layer at borderline, which remains relatively thin and confined near the wall. This confinement creates a steeper temperature gradient at the surface, leading to more efficient heat transfer (higher Nusselt number) but, crucially, results in a sharper decay of the thermal profile $\left\{ {\theta \left( \eta \right)} \right\}$ away from the wall; the influence of the wall temperature penetrates only a short distance into the fluid, causing the non-dimensional temperature to drop more quickly with distance compared to low-$\left( {\Pr } \right)$ fluids like liquid metals, where heat diffuses far into the stream. The impression of Brownian motion parameter $\left( {Nb} \right)$ on $\left\{ {\theta \left( \eta \right)} \right\}$ is explained in Fig. 14. With augmentation in $\left( {Nb} \right)$ there is a rise in $\left\{ {\theta \left( \eta \right)} \right\}$. Actually, Brownian motion parameter captures the effect of the random, thermal agitation of nanoparticles suspended in a base fluid. A physical interpretation of the rise in $\left\{ {\theta \left( \eta \right)} \right\}$ with an intensification in this parameter is that enhanced Brownian motion intensifies the micro-scale mixing and energy exchange between the nanoparticles and the surrounding fluid molecules. This vigorous, chaotic motion disrupts the relatively stagnant thermal layer at borderline near the heated surface, effectively augmenting the effective thermal conductivity of the fluid and facilitating a more efficient transport of heat away from the wall. Consequently, the thermal energy penetrates further into the bulk fluid, causing a fuller temperature profile; instead of a steep decay near the wall, the elevated Brownian motion promotes a more uniform thermal distribution, leading to the non-dimensional temperature to remain higher across the boundary layer. The impression of thermo-phoresis parameter $\left( {Nt} \right)$ on $\left\{ {\theta \left( \eta \right)} \right\}$ is explained in Fig. 15. With augmentation in $\left( {Nt} \right)$ there is a rise in $\left\{ {\theta \left( \eta \right)} \right\}$. This parameter quantifies the phenomenon where suspended nanoparticles migrate from regions of higher temperature toward regions of lower temperature under the influence of a temperature gradient. A physical interpretation of the rise in $\left\{ {\theta \left( \eta \right)} \right\}$ with an intensification in this parameter is that enhanced thermophoresis drives a greater flux of hot nanoparticles away from the heated surface and deeper into the colder bulk fluid. As these energetic particles travel outward, they carry thermal energy with them, effectively augmenting the convective heat transport within the boundary layer. This nanoparticle-mediated energy transfer reduces the steepness of the near-wall temperature gradient, allowing heat to penetrate further into the fluid flowing on the surface. Consequently, the thermal profiles $\left\{ {\theta \left( \eta \right)} \right\}$ rise, indicating a more distributed and efficient thermal transport compared to scenarios with weaker thermophoretic effects. The impression of heat source/sink $\left( Q \right)$ on $\left\{ {\theta \left( \eta \right)} \right\}$ is explained in Fig. 16. With augmentation in $\left( Q \right)$ there is a rise in $\left\{ {\theta \left( \eta \right)} \right\}$. In fluid mechanics, the heat source/sink parameter represents internal volumetric generation or absorption of thermal energy within the fluid. A physical interpretation of the rise in $\left\{ {\theta \left( \eta \right)} \right\}$ with an intensification in this parameter specifically for a heat source is that the fluid now gains thermal energy internally throughout the domain rather than solely relying on conduction and convection from the bounding surface. This volumetric energy addition acts as a distributed heating mechanism that continuously elevates the fluid temperature at every point within the flow field. As the heat source strengthens, the near-wall fluid becomes additionally energized from within, reducing the efficacy of wall cooling and weakening the temperature gradient at the surface. Consequently, the temperature profiles $\left\{ {\theta \left( \eta \right)} \right\}$ rise across the entire domain on the surface of the sheet, reflecting a diminished capacity to dissipate the internally generated heat relative to the energy being introduced. The impression of thermal relaxation time parameter $\left( \delta \right)$ on $\left\{ {\theta \left( \eta \right)} \right\}$ is explained in Fig. 16. With augmentation in $\left( \delta \right)$ there is decline in $\left\{ {\theta \left( \eta \right)} \right\}$. From a fluid mechanics perspective, the decline in $\left\{ {\theta \left( \eta \right)} \right\}$ with an increasing $\left( \delta \right)$ reflects the transition from a Fourier (diffusive) heat transfer regime to a non-Fourier regime, where the finite speed of thermal propagation becomes substantial. Physically, this parameter explains the time lag between the establishment of the temperature gradient and the onset of heat flow. When this parameter is large, the thermal inertia of the medium is substantial, meaning the internal energy of the fluid particles cannot adjust instantaneously to the surrounding thermal field. Actually, the thermal relaxation time parameter $\left( \delta \right)$, which characterizes the transition from Fourier to non-Fourier heat conduction and introduces a thermal memory effect in the system. This memory effect implies that heat flux does not respond instantaneously to temperature gradients, but rather retains a dependence on the prior thermal history of the material. From an application perspective, this behavior can be exploited in high-temperature manufacturing processes such as metal forming, additive manufacturing, and polymer extrusion, where rapid thermal loading often leads to non-uniform temperature distributions and thermal stresses. The presence of thermal relaxation delays heat propagation, thereby smoothing abrupt thermal gradients and reducing localized thermal shocks. As a result, the memory effect associated with $\left( \delta \right)$ can help in mitigating thermal distortion and preventing material warping during rapid heating and cooling cycles. This additional discussion has been included to highlight the practical relevance of the non-Fourier heat conduction model in industrial thermal processing.

### Table discussion

Table 1 summarizes the numerical performance for all three ANN configurations, presenting the Mean Squared Error (MSE) for training, validation, and testing that are the key indicators of predictive accuracy and generalization. It also records essential training dynamics, including network performance, gradient, and the mu parameter, at specific epochs (134, 205, and 175). Tracking the evolution of these values offers meaningful insight into the network’s convergence behavior. This information is instrumental in refining both the architectural design and training hyper-parameters, ensuring a well-optimized model with reliable predictive capability. Table 2 examines the effects of various parameters on skin friction $\left\{ {\sqrt {R{e_x}} {\mkern 1mu} {C_{{f_x}}}} \right\}$ and Nusselt number $\left\{ {N{u_x}/\sqrt {R{e_x}} } \right\}$. From this table it is obvious that with growth in magnetic parameter $\left( M \right)$, porosity parameter $\left( K \right)$, Darcy Forchheimer factor $\left( {Fr} \right)$ and Weissenberg number $\left( {We} \right)$ there is decline in skin friction $\left\{ {\sqrt {R{e_x}} {\mkern 1mu} {C_{{f_x}}}} \right\}$. An intensification in $\left( M \right)$ strengthens the Lorentz force, which opposes fluid motion and reduces velocity gradients at the wall, lowering $\left\{ {\sqrt {R{e_x}} {\mkern 1mu} {C_{{f_x}}}} \right\}$. Higher $\left( K \right)$ and $\left( {Fr} \right)$ intensify resistance within the porous medium due to increased solid–fluid interactions and inertial effects, further suppressing near-wall momentum. A larger $\left( {We} \right)$ reflects enhanced elasticity in viscoelastic fluids, which reduces shear stress by storing deformation energy rather than dissipating it through viscous effects. Collectively, these factors diminish the frictional drag exerted by the fluid on the boundary. Table 2 further explains with growth radiation parameter $\left( {Rd} \right)$, heat source parameter $\left( Q \right)$ and Eckert number $\left( {Ec} \right)$ there is augmentation in Nusselt number $\left\{ {N{u_x}/\sqrt {R{e_x}} } \right\}$ while a decline in it with intensification in Prandtl number $\left( {\Pr } \right)$. An intensification in $\left( {Rd} \right)$ enhances thermal diffusion through photon transport, augmenting heat transfer from the surface and thus raising $\left\{ {N{u_x}/\sqrt {R{e_x}} } \right\}$. A higher $\left( Q \right)$ supplies additional energy to the fluid near the wall, steepening the temperature gradient and further improving heat transfer. Greater $\left( {Ec} \right)$ signifies intensified viscous dissipation, which acts as an internal heat source, elevating wall heat flux. Conversely, a larger $\left( {\Pr } \right)$ thickens the thermal boundary layer relative to the momentum boundary layer, reducing the temperature gradient at the wall and consequently diminishing $\left\{ {N{u_x}/\sqrt {R{e_x}} } \right\}$. Actually, the Prandtl number represents the ratio of momentum diffusivity to thermal diffusivity, indicating the relative thickness of the velocity and thermal boundary layers. As $\left( {\Pr } \right)$ rises, the thermal diffusivity decreases compared to momentum diffusivity, which reduces heat conduction within the fluid and weakens thermal transport away from the surface. Consequently, the thermal boundary layer becomes thinner, leading to a reduction in the temperature gradient at the wall and hence a decrease in the Nusselt number, which measures the rate of heat transfer. This behavior is further influenced by the limited ability of thermal energy to diffuse rapidly in high-$\left( {\Pr } \right)$ fluids, causing heat to remain closer to the wall region rather than being efficiently transported into the fluid bulk.

**Table 1:** Numerous results of case-1 of each scenario for the REF flow model

Cases	Epoch	Time (s)	Mean squared error			Performance	Gradient	Mu
			Validation	Test	Train			
*S*_1_ & *C*_1_	134	08	0.73 × 10^−9^	0.56 × 10^−7^	0.42 × 10^−7^	0.78 × 10^−8^	0.16 × 10^−7^	0.1 × 10^−7^
*S*_2_ & *C*_1_	205	15	0.44 × 10^−9^	0.42 × 10^−9^	0.29 × 10^−7^	0.32 × 10^−9^	0.54 × 10^−7^	0.1 × 10^−9^
*S*_3_ & *C*_1_	275	11	0.32 × 10^−5^	0.87 × 10^−5^	0.82 × 10^−5^	0.42 × 10^−6^	0.24 × 10^−5^	0.1 × 10^−5^

**Table 2:** Numerous results for skin friction $\left\{ {\sqrt {R{e_x}} {\mkern 1mu} {C_{{f_x}}}} \right\}$ and Nusselt number $\left\{ {N{u_x}/\sqrt {R{e_x}} } \right\}$ against variations in various parameters

M	K	We	Fr	Rd	Q	Pr	Ec	$\sqrt {R{e_x}} {\mkern 1mu} {C_{{f_x}}}$	$N{u_x}/\sqrt {R{e_x}}$
0.5	1.0	0.0	0.5	1.0	0.3	0.5	1.0	1.618347	–
1.0								1.604377	–
1.5								1.546533	–
	1.0							1.446257	–
	2.0							1.332239	–
	3.0							1.329379	–
		0.0						1.545944	–
		1.0						1.418579	–
		2.0						1.243706	–
			0.5					1.392501	–
			1.0					1.364635	–
			1.5					1.302131	–
				1.0				–	0.1566167
				1.5				–	0.2414621
				2.0				–	0.3004845
					0.3			–	0.5258721
					0.5			–	0.6204456
					0.7			–	0.7087321
						0.5		–	0.4845821
						0.7		–	0.3213145
						0.9		–	0.2984223
							1.0	–	0.6050846
							2.0	–	0.7561734
							3.0	–	0.7911510

### Sensitivity analysis and network optimization

A statistical reliability and mathematical optimization of the proposed multi-layer Cascade Forward Neural Network was carried out. The network architecture is composed of two cascading hidden layers, the number of nodes in which is to be varied systematically in order to ensure the absolute minimum Mean Squared Error (MSE) is obtained. To observe the convergence behavior and to avoid the obstacles of overfitting or high computation costs associated with different architectural permutations, from under-parametrized to over-parametrized network grid, were tested. The detailed benchmarking parameters are shown in Table 3, and they include the Training, Validation, and Testing MSE for four different neuron layer distributions.

**Table 3:** Quantitative sensitivity analysis for hidden layer neuron configuration optimization

Case	Hidden layer 1	Hidden layer 2	Training	Validation	Testing
	Neurons		MSE		
1	2	2	1.54 × 10^− 5^	2.10 × 10^− 5^	1.98 × 10^− 5^
2 (Optimal)	3	3	0.29 × 10^− 7^	0.44 × 10^− 9^	0.42 × 10^− 6^
3	4	4	3.12 × 10^− 6^	4.85 × 10^− 6^	5.21 × 10^− 6^
4	5	5	8.65 × 10^− 6^	1.23 × 10^− 5^	1.11 × 10^− 5^

**Fig. 18:**
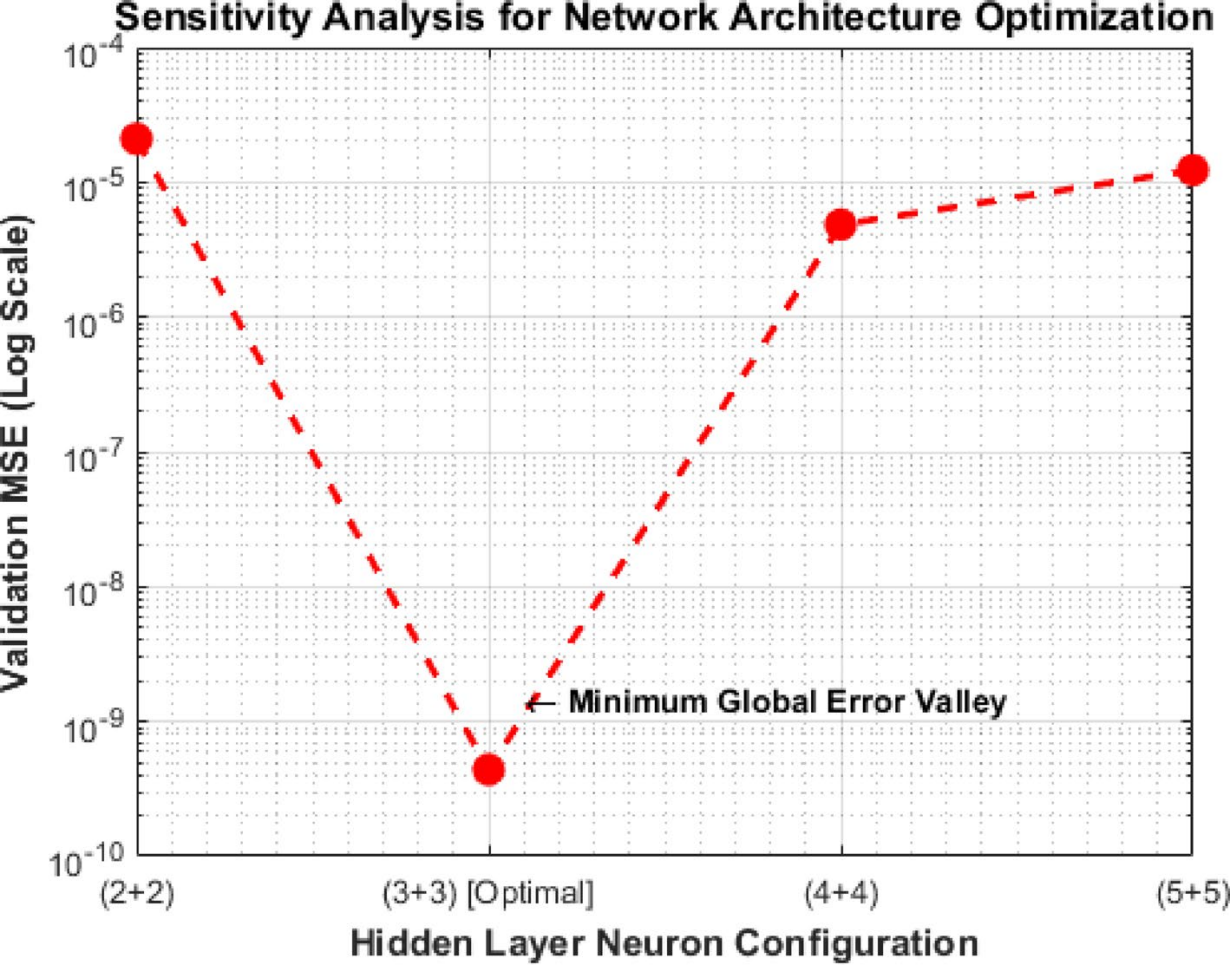
Sensitivity analysis curve demonstrating the validation MSE profiles across distinct hidden layer neuron configurations

The graphical progression of the validation error profiles across the tested architectures is shown in Fig. 18.

The quantitative results show that the (2 + 2) architecture does not capture the nonlinear dynamics of the magnetized dissipative fluid flow because it is under-fitting, but when the layers are increased to (4 + 4) and (5 + 5), it actually deteriorates. This error elevation in the higher neuron blocks is associated with overfitting tendencies, so the model will contain secondary numerical noise instead of the main flow profiles. The (3 + 3) configuration gives the steepest convergence drop and reaches the minimum global validation of 0.44 × 10^− 9^. Mathematically, it is shown that this physical model has the optimum structure when exactly 3 neurons are used for each cascading hidden layer with standard *R* = 1 correlation constraint.

## Conclusions

This work investigates the magnetohydrodynamic flow of a dissipative Ree-Eyring fluid on a permeable stretching surface, subjected to an inclined magnetic field relative to the direction of fluid motion. Thermal transport is analyzed using the Cattaneo-Christov heat flux model, which accounts for thermal relaxation effects absent in the classical Fourier formulation. The flow regime further incorporates the influence of a Darcy-Forchheimer porous medium, introducing both linear and quadratic drag contributions to the momentum equation. The main equations have initially been evaluated numerically through bvp4c approach in dimensionless form. The dataset generated through the bvp4c numerical scheme is subsequently employed to implement the artificial neural network (ANN) methodology. After comprehensive analysis of the work, it has deducted that:

- A ten-neuron hidden layer balances model complexity to prevent under-fitting and over-fitting, with optimal MSE convergence achieved at epochs 134, 205, and 275 across the three scenarios.
- Error histograms and fitness evaluation confirm solution stability, progressive improvement, and close alignment between predicted and expected values.
- Regression correlation coefficients (R) near unity across all datasets validate exceptional predictive accuracy, establishing the ANN as a dependable tool for analyzing coupled transport phenomena.
- With growth in Weissenberg number $\left( {We} \right)$, magnetic parameter $\left( M \right)$, Darcy Forchheimer factor $\left( {Fr} \right)$ and porosity parameter $\left( K \right)$ there is decline in velocity $\left\{ {f^{\prime}\left( \eta \right)} \right\}$.
- Thermal distribution $\left\{ {\theta \left( \eta \right)} \right\}$ augmented with growth in radiation parameter $\left( {Rd} \right)$, Brownian motion parameter $\left( {Nb} \right)$, thermo-phoresis parameter $\left( {Nt} \right)$, heat source/sink factor $\left( Q \right)$while declined with higher Prandtl number $\left( {\Pr } \right)$ and thermal relaxation time parameter $\left( \delta \right)$.
- With growth in magnetic parameter $\left( M \right)$, porosity parameter $\left( K \right)$, Darcy Forchheimer factor $\left( {Fr} \right)$ and Weissenberg number $\left( {We} \right)$there is decline in skin friction $\left\{ {\sqrt {R{e_x}} {\mkern 1mu} {C_{{f_x}}}} \right\}$.
- For higher radiation parameter $\left( {Rd} \right)$, heat source parameter $\left( Q \right)$ and Eckert number $\left( {Ec} \right)$ there is augmentation in Nusselt number $\left\{ {N{u_x}/\sqrt {R{e_x}} } \right\}$ while a decline in it with intensification in Prandtl number $\left( {\Pr } \right)$.

### Limitation of current work

The current study is primarily based on a theoretical investigation and provides valuable analytical insights into the considered problem. However, a key limitation of the present work is the absence of experimental validation to support and verify the theoretical findings. Therefore, future work would focus on experimental investigation to complement the theoretical framework and to establish stronger agreement between theoretical predictions and real-world behavior.

## Data Availability

The data that support the findings of this study are available from the corresponding author upon reasonable request.
